# Anticancer Potential of the Principal Constituent of Piper nigrum, Piperine: A Comprehensive Review

**DOI:** 10.7759/cureus.54425

**Published:** 2024-02-18

**Authors:** Vidhya Rekha Umapathy, Anandhi Dhanavel, R Kesavan, Prabhu Manickam Natarajan, Bhuminathan S, Vijayalakshmi P

**Affiliations:** 1 Public Health Dentistry, Meenakshi Ammal Dental College and Hospital, Meenakshi Academy of Higher Education and Research (MAHER), Chennai, IND; 2 Biochemistry, Meenakshi Academy of Higher Education and Research (MAHER), Chennai, IND; 3 Public Health Dentistry, Thai Moogambigai Dental College and Hospital, Chennai, IND; 4 Clinical Sciences/Periodontics, College of Dentistry, Ajman University, Ajman, ARE; 5 Public Health Dentistry, Sree Balaji Dental College & Hospital, Chennai, IND; 6 Biotechnology, Holy Cross College (Autonomous) Tiruchirappalli, Tiruchirappalli, IND

**Keywords:** review, pepper, anticancer activity, piperine, piper nigrum

## Abstract

Black pepper’s main component, piperine, is a phytochemical that gives the spice its distinctively pungent flavor, which has made it a staple in human diets for decades and a widely used food item. In addition to its use as a culinary component and preservation agent, it is also employed in traditional medicine for a diverse range of objectives, a practice that has been substantiated by existing scientific investigations on its physiological impacts in the majority of instances. Piperine contains various bioactive effects, such as antibacterial activity, in addition to several physiological benefits that could help overall human health, such as immunomodulatory, hepatoprotective, antioxidant, antimetastatic, anticancer, and many more properties that have been established. Clinical trials revealed that this phytochemical has exceptional antioxidant, anticancer, and drug availability-enhancing properties, as well as immunomodulatory potential. The different components of evidence indicate the therapeutic potential of piperine and underscore the importance of incorporating it into both broad health-promoting interventions and supplementary treatment pharmaceutical formulations. This inclusion can enhance the bioavailability of other therapeutic medications, including those used in chemotherapy.

## Introduction and background

Piperine, an alkaloid compound, is responsible for the distinct flavor characteristics observed in several pepper species. Additionally, it was detected in several other botanical species, including *Rhododendron faurie*, *Vicoa indica*, and *Anethum sowa*, among others [1]. Piperine content varies from 2% to 9% in *Piper nigrum* L. based on ecologic factors such as climate and/or provenance, as well as the growing environment [2]. *P. nigrum* L., often known as black pepper, is the most extensively utilized species of pepper with a global presence. The substance in question serves as a culinary seasoning and possesses significant therapeutic properties. Due to its prominent status within the Ayurvedic system of medicine, black pepper has been historically incorporated as a constituent of the “tricatu” module, along with long pepper and ginger. This traditional usage may be traced back to ancient times, when these three ingredients were combined in equal proportions [3]. Pepper has historically been employed for the therapeutic management of several ailments, including febrile conditions, gastrointestinal disorders, neurological disorders, and respiratory afflictions such as asthma and chronic bronchitis. In traditional Chinese medicine, black pepper is employed for its therapeutic properties in addressing many health conditions, such as headaches, rheumatism, strep throat, and influenza, and promoting enhanced blood circulation. The distinctive taste and aroma of black pepper essential oil can be ascribed to the existence of certain components inside the oil. These chemicals can be present in the fruit at a concentration of up to 3.5% [4]. This essential oil contains a variety of molecules, including sabinene, pinene, etc. Piperine, also known as piperoylpiperidine (C17H19NO3), is a prominent alkaloid found in pepper that contributes to its strong flavor and exhibits several pharmacological properties with strong antioxidant properties. Research on the bioactivities of piperine has uncovered a diverse array of physiological effects, including but not limited to antihypertensive, antiaggregant, antioxidant, anticancer, antispasmodic, antiasthmatic, depressive, and anxiolytic properties [5]. Piperine demonstrates multiple biological functions and possesses the capacity to increase medicine absorption, hence increasing its therapeutic effectiveness [6]. In addition to its various health advantages, piperine has been employed as a dietary component for thousands of years, serving as the primary constituent of the widely recognized spice known as pepper. Moreover, its consumption does not pose any discernible health hazards. The genotoxicity of piperine was evaluated by Ames assays, and micronucleus tests [7] have been reported in other research, indicating that it is safe to consume. This review covers all of the existing information on piperine’s anticancer properties.

## Review

Piperine’s significance in food

The major dietary alkaloid in the Piperaceae family, found in both *P. nigrum* L. (black pepper) and *Piper longum* (long pepper), is piperine (1-piperoylpiperidine) [8]. Black pepper, commonly referred to as the “king of spices” in Indian medicine, has been employed for the treatment of respiratory and gastrointestinal ailments [9]. In 1819, Hans Christian Ørsted successfully achieved the isolation of piperine from pepper extracts, as documented by Gorgani et al. Alkaloids are a class of chemical compounds derived from natural sources, characterized by a cyclic structure and the presence of a central nitrogen atom. The majority of heterocyclic rings consist primarily of nitrogen atoms [10]. Two alkaloids that have obtained the FDA approval include camptothecin, a recognized inhibitor of topoisomerase I, and vinblastine, which interacts with tubulin and induces mitotic catastrophe. For instance, vinblastine has been licensed by the FDA [11]. Piperine has shown preventive effects against several cancer-causing substances, according to a 2012 study by Lu et al.

Chemopreventive mechanisms of piperine

Breast cancer prevention drugs such as raloxifene and tamoxifen, as well as medications that can treat preneoplastic skin lesions, were all developed in the early stages of human history to address the growing need for cancer chemotherapy [12]. The aforementioned qualities span a range of cellular effects, including the initiation of apoptotic signaling pathways, suppression of cell growth, halting of the cell cycle, preservation of redox equilibrium, modification of endoplasmic reticulum (ER) stress and autophagy, and promotion of detoxifying enzyme activity [13,14]. These methods of action offer convincing proof of piperine’s essential role in cancer chemoprevention.

Anticancer properties of piperine: an in vitro and in vivo analysis

Piperine, a prominent bioactive constituent of *P. nigrum*, is present within this botanical species. It is in charge of giving pepper its recognizable pungent flavor. The perception of pungency arises from the stimulation of transient receptor potential cation channels for vanilloid. Typically, piperine is extracted and refined from pepper fruits and roots, but because of its extensive biological potential, innovative approaches to its production, including the utilization of endophytes, have been developed. One potential source of metabolites found in the host plants, such as piperine, is endophytic fungi isolated from *P. nigrum* or *P. longum*. By optimizing their growing conditions, it may be possible to biosynthesize this alkaloid on a large scale [15]. Research on piperine’s anticancer effects has focused on both its anticancer activity alone and its anticancer activity in combination with other anticancer medicines. Piperine suppresses cancer cell proliferation and increases apoptosis [16].

Induction of cell death

**Figure 1:**
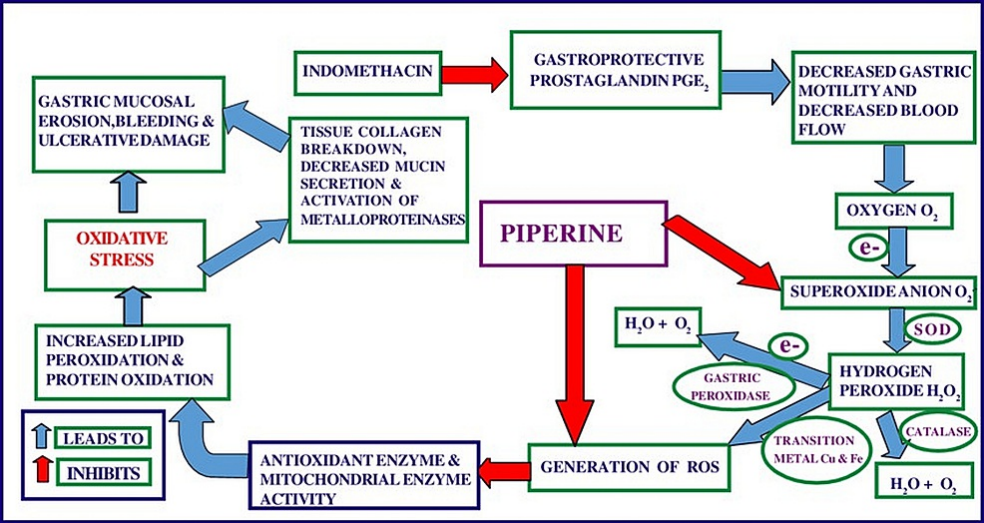
Mechanism of action of piperine

Numerous studies have provided evidence supporting the capacity of piperine to induce the activation of caspase-3, a caspase that operates downstream in the signaling pathway [16]. Additionally, piperine has been shown to stimulate the activity of poly(ADP-ribose) polymerase in various cancer cell lines, including breast [17], prostate [18], colon [19], melanoma [20], and ovarian [21]. Several scholarly articles have documented the ability of piperine to activate apoptotic final effectors such as caspase-3. However, there is a limited amount of research that has investigated the upstream targets implicated in this process. The upregulation of survivin is observed in human malignancies, wherein it functions in the modulation of cytokinesis and the development of the cell cycle. Surprisingly, Yaffe et al. [19] showed that piperine boosted pro-caspase-3 and pro-caspase-7 activation while inhibiting survivin in colon cancer cells. The suppressive effects of YM-155, a survivin suppressor, on colon cancer cells were seen to diminish the proapoptotic activity of piperine. This shows that survivin expression strongly affects piperine's anticancer actions. Piperine decreased survivin synthesis and p65 phosphorylation in breast cancer cells from human and animal sources, supporting Abdelhamed et al.’s study [22]. This observation implies that the reduction of survivin expression with piperine administration induces apoptotic responses in several types of cancer (Figure 1).

Based on the research conducted by Greenshields et al. [23], it has been observed that piperine exhibits the ability to inhibit the activation of the intrinsic apoptotic pathway in breast cancer cell lines characterized by basal human epidermal growth factor receptor 2 (HER2) expression. This inhibition is achieved through the modulation of Smac/DIABLO. In melanoma cells, Yoo et al. [24] validated the regulation of the proapoptotic pathway by piperine. The current study found that piperine inhibited human X-linked IAP (XIAP), a protein that prevents apoptosis. XIAP is well acknowledged as one of the most thoroughly investigated IAP proteins. In the context of apoptosis, as examined by Fulda et al. [16], it is demonstrated that piperine possesses the capacity to regulate the interaction between Smac/DIABLO and XIAP. The regulation outlined in the statement leads to the translocation of caspase-9 from XIAP, hence facilitating the assembly of the apoptosome complex, including cytochrome c, Apaf-1, and caspase-9.

Piperine’s intrinsically active apoptotic-inducing abilities have been demonstrated in a variety of cancer cell lines [25]. Piperine controls a lot of proteins in the Bcl-2 family, which includes Bax and Bcl-2. A study by Fofaria et al. found that piperine could stop the action of Bid, an unsheaved BH3 interaction domain death agonist, in cells with melanoma [20]. The crucial connection bridging the intrinsic and extrinsic pathways can be bid. This is because cleavage of Bid by caspase-8 results in the release of cytochrome c. As a result, this cytochrome c release causes the mitochondria’s caspases to activate [16]. In contrast, piperine has not demonstrated the ability to induce caspase-8 activation in any studies conducted. In addition, the administration of specific caspase-3 and caspase-9 inhibitors before exposing ovarian cancer cells to piperine resulted in the prevention of piperine-induced apoptosis. However, the presence of a caspase-8 inhibitor did not have any impact on the proapoptotic effects of piperine, as demonstrated by Si et al. [21]. The findings of this investigation suggest that the extrinsic pathway was not activated in the given environment.

In melanoma and lung cancer cells, piperine boosted p53 expression [26]. Greenshields et al., on the other hand, demonstrated that piperine’s anticancer effects were independent of the presence of a functional p53. According to Yaffe et al. [19], piperine reduced cell proliferation in p53/HCT116 cells, and colon cancer patients also saw a similar effect. Because p53 is frequently mutated or nonfunctional in a variety of malignancies [27], piperine’s proapoptotic activity independent of p53 may be clinically relevant. In human cancers, like breast tumors, fatty acid synthase expression is strongly correlated with aggressiveness and cell cycle progression [28,29]. It is important to note that piperine has shown the capacity to inhibit the growth of fatty acid synthase by inhibiting mature sterol regulatory element-binding protein-1 and extracellular signal-regulated kinase 1/2. Consequently, this caused breast tumor cells that overexpressed HER2 to die.

G-quadruplex structures are non-standard DNA shapes that form when G-quartets are arranged in a square, flat pattern. Most G-quadruplex structures are made in telomeres and oncogene regulatory regions, mainly c-myc, when cells divide. These structures are of utmost importance in the preservation of shortened telomeres and the facilitation of genomic instability, hence promoting tumor growth [30]. The potential of piperine to induce apoptosis in cancer cells may be linked to the stability of the G-quadruplex structure, as shown by Tawani et al. [31], thereby presenting a plausible mechanism.

Numerous natural substances have anticancer properties via their autophagy-regulating properties. Autophagy is a two-edged sword that could either repress or accelerate carcinogenesis in cancer cells. In conjunction with apoptosis, prior studies have established that piperine exhibits the capacity to elicit autophagy in prostate cancer [32]. Androgen proteins are very important for prostate cancer to grow and stay alive, especially in the early stages. According to Samykutty et al. [18], the administration of androgen ablation therapy leads to the remission of cancer. Regardless of the precise method by which piperine elicits cellular responses, it is commonly recognized among researchers that androgen-dependent cells, namely LNCaP cells, have heightened vulnerability to the cancer-fighting properties of piperine in contrast to prostate cancer cells (PC3 and DU145) [18].

The process of inducing cell cycle arrest

Piperine has demonstrated the ability to impede the multiplication of cancer cells via multiple pathways. Piperine exhibits the capacity to halt the progression of the cell cycle at many phases, including G0/G1, S, or G2/M, depending on the particular cell type and the degree of tumor activity. The compound piperine has been observed to demonstrate inhibitory effects on the S phase of the cell cycle, particularly in leukemia cells [33-37]. Conversely, in the case of colon, melanoma [20], and prostate tumor cells [18], piperine was observed to inhibit the G1 level of the cell cycle. The G1 phase is characterized by a decline in cyclin D concentration and an upregulation of p21 expression. The term “P21” refers to a classification of chemical substances that are acknowledged as inhibitors of cyclin-dependent kinases (CDKs) [35]. Moreover, it has been established that the existence of P21 leads to the suppression of cyclin A and B functions, both of which play a crucial part in advancing through the S and G2/M phases, correspondingly [28].

Furthermore, the elevation of p27 expression was observed upon the administration of piperine, in conjunction with the overexpression of p21. It is well established that p27 exerts various impacts on the advancement of the cell cycle. One example that demonstrates this phenomenon is the suppression of CDK4-cyclin D and CDK6-cyclin D activity by p27, leading to the cessation of the cell cycle, particularly in the G1 phase. Fofaria et al. [24] conducted a study that provided evidence for the association between the induction of G1 arrest and DNA damage caused by piperine’s prooxidant characteristics. This DNA damage subsequently led to the activation of Chk1, a checkpoint kinase. The study demonstrated the ability of melanoma cells to safeguard themselves against piperine-induced cell cycle arrest by either employing a particular inhibitor to suppress Chk1 activity or utilizing siRNA. This finding suggests that the phosphorylation of Chk1 is of utmost importance in the G1 inhibition induced by piperine. Therapeutic injection of piperine inhibited osteosarcoma cell proliferation during the G2/M phase, which is linked to Chk2 phosphorylation. The aforementioned tests provide a detailed chronology of the effects induced by piperine, which include the activation of the ATR/Chk/p53/p21 signaling pathway, subsequent cellular growth inhibition, and ultimately, apoptosis.

When examining the data collectively, it becomes evident that the cell cycle regulators targeted by piperine exhibit a high degree of specificity toward particular types of tumor cells. Moreover, these regulatory factors play a pivotal role in regulating the precise phase of the cellular cycle during which the disruption takes place.

The utilization of three-dimensional (3D) cell cultures presents a prospective connection between monolayer cell culture and animal experiments, as they exhibit a higher resemblance to in vivo tumor characteristics as compared to two-dimensional cell cultures. In recent decades, notable advancements have been made in the field of 3D model development. Numerous techniques have been suggested for creating 3D in vitro cancer models. One notable approach highlighted in the 2017 study conducted by Lv et al. [38] involves the use of multicellular tumor spheroids. In research conducted by Greenshields et al. [23], it was observed that piperine demonstrated inhibitory properties toward the growth of mammospheres, which are spherical structures commonly linked to breast and colon cancer. The inhibitory effect of piperine on the proliferation of mammospheres expressing typical markers of cancer stem cells (CSCs) was found to be significant, as reported in a study conducted by Cioce et al. [39]. Nevertheless, Serrano-Novillo et al. [40] reported that the antiproliferative impact of piperine was much less pronounced in breast cancer monolayer cell cultures.

The maintenance of CSC self-renewal is influenced by various aberrant pathways, such as Hedgehog, Notch, and Wnt, as reported by Liu et al. [41]. The application of piperine at concentrations ranging from 5 to 10 M resulted in a decrease in the self-renewal capacity of breast CSCs. This effect was attributed to the inhibition of the Wnt signaling pathway. The results of this study demonstrate that piperine has a crucial role in regulating the aberrant proliferation of CSCs, which is a significant distinguishing feature observed in different types of malignancies.

Inhibition of angiogenic progression

The process of angiogenesis is an essential component in the progression of malignancies since it facilitates the expansion of tumors [42,43]. Consequently, antiangiogenic drugs have been incorporated into the clinical armamentarium for combating cancer. The study conducted by Doucette et al. investigated the impact of piperine on several stages of the angiogenesis process. The study authors presented empirical evidence demonstrating that piperine exhibited inhibitory properties on the proliferation of human umbilical vein endothelial cells, specifically during the G1/S transition phase, without inducing any harmful effects. Subsequently, the researchers proceeded to create ex vivo and in vivo models to investigate the impact of piperine on blood vessel sprouting produced by collagen. The rat aorta angiogenesis model and the chick embryo chorioallantoic membrane assay were employed to achieve this.

In vivo experiments

The current body of research on the anticancer properties of piperine is primarily limited to in vitro investigations, with a notable lack of studies conducted on animal models to further understand its mechanisms of action. The inaugural presentation by Pradeep et al. [44] almost 20 years ago showcased in vivo evidence of piperine’s capacity to hinder lung metastasis induced by melanoma cells in C57BL/6 mice. Lung metastasis was observed in all animals that received an injection of B16F-10 melanoma cells into the lateral tail vein. Lung fibrosis is a pathological condition that occurs due to the presence of lung metastases. The degree of pulmonary fibrosis linked to metastasis is correlated with the abundance of collagen hydroxyproline accumulated within the alveoli of the lungs. The production of a significant amount of uronic acid by tumor cells, caused by the oxidation of sugar derivatives known as aldoses, leads to the buildup of hydroxyproline [44]. The conversion of pro-hydroxyproline to hydroxyproline is facilitated by uronic acid through the generation of glucuronic acid lactone. Hexosamine, a sugar derivative, has been identified as a constituent present in tumor cells. According to Zhang et al. [45], the synthesis of sialic acid is essential as it serves as a constituent of the outer layer of cancerous cells. The concentration of sialic acid has been found to have a strong correlation with the occurrence of metastasis and an unfavorable prognosis. Rapidly proliferating tumors necessitate intracellular glutathione (GSH) for energy acquisition and the sustenance of tumor growth and dispersion. The enzyme gamma-glutamyl transferase (GGT) facilitates the intracellular synthesis of GSH through the gamma-glutamyl cycle. Consequently, GGT can be utilized as an appropriate indicator for both cell proliferation and metastasis [46]. Administering piperine therapy to mice at a dosage of 200 mol/kg body weight via intraperitoneal injection for a duration of 10 days resulted in a significant reduction in tumor nodule growth and an extended life span in the mice treated with piperine, increasing from 31 to 90 days. Moreover, the injection of piperine led to a decrease in the levels of diverse biochemical markers commonly linked to metastatic lung tissue. The indicators under consideration encompass collagen hydroxyproline, lung uronic acid, and hexosamine. Moreover, the administration of piperine resulted in a significant reduction in the levels of sialic acid and GGT in the serum of mice with tumors. The biomarkers have been observed to exhibit significant expression levels in animals with tumors. Despite the stated extension of mice’s life span after the 90-day study period, substantial flaws emerged as a result of the absence of an evaluation regarding the safety profile of piperine in this investigation.

The current review has examined the possible anticancer properties of piperine in Wistar albino rats that were generated with hepatocarcinoma using diethylnitrosamine (DEN), a liver carcinogen recognized for its significant genotoxic effects in rats. In rats subjected to DEN therapy, the administration of piperine at a dosage of 5 mg/kg body weight led to a notable reduction in liver enzyme markers commonly linked with toxicity, including aspartate transaminase, alkaline phosphatase, and alanine transaminase. Furthermore, piperine supplementation exhibited a positive effect on the structural integrity of the liver in these rats. In addition, the administration of piperine led to the suppression of tumor growth through the downregulation of Ki67 expression, a marker closely linked to cellular proliferation. Furthermore, there was a simultaneous rise in the proportion of apoptotic cells, as found in mice subjected to DEN treatment. Intriguingly, the combined administration of piperine and EUK 134, an antioxidant compound that emulates the catalytic function of catalase, demonstrated notable effectiveness in suppressing the anticancer properties of the phytochemical in rats afflicted with hepatocarcinoma induced by DEN, as observed in the aforementioned study. The study conducted by Gunasekaran et al. revealed that in rats suffering from DEN-induced hepatocarcinoma, the administration of piperine in combination with EUK 134, an antioxidant compound that mimics the catalytic function of catalase, was notably effective in suppressing the phytochemical's anticancer properties [47]. Hence, our investigation has provided in vivo evidence suggesting that the primary mechanism underlying the anticancer effects of piperine is likely associated with its prooxidant activity.

Furthermore, the administration of piperine at a dosage of 100 mg/kg/die via intraperitoneal injection over one month exhibited noteworthy suppression of tumor growth in xenotransplanted nude mice harboring either androgen-dependent (LCNaP) or androgen-independent (DU145) prostate cancer cells. The research study documented a reduction in tumor growth by 72% in mice that were xenotransplanted with LNCaP cells and a drop of 41% in animals that were xenotransplanted with DU145 cells. The present work provides evidence supporting the effectiveness of piperine in suppressing the growth of androgen-dependent prostate cancer cells in a laboratory setting. Moreover, the anticancer efficacy of piperine was validated in nude mice through the use of xenotransplantation with LCNaP cells. The mice in this study had oral administration of a therapy by gavage at a dosage of 10 mg/kg body weight for one month. The primary focus of this study was on cancers that are dependent on androgens.

Moreover, piperine has demonstrated anticancer activity in in vivo models of breast cancer. In a study conducted by Talib et al. [48], BALB mice were utilized as experimental subjects. These mice were implanted with mouse mammary EMT6/P cancer cells. The administration of piperine was carried out via intraperitoneal feeding, with a dosage of 25 mg/kg/day. This treatment regimen was maintained for 14 days. The findings demonstrate a statistically significant decrease in tumor growth, indicating a drop of 15% when compared to the negative control group. On the other hand, the negative control group demonstrated a significant 79% rise in tumor size. Moreover, in a murine model of breast cancer, the intratumoral injection of piperine (at doses of 2.5 or 5 mg/kg, administered every three days for a total of three injections) exhibited a dose-dependent suppression of primary 4T1 tumor growth. The anticancer effects identified in this study were attributed to the substance’s proapoptotic and antiproliferative properties, as evidenced by its impact on murine tumor tissues [49]. In a study conducted by Abdelhamed et al. [22], it was found that the administration of piperine at a dosage of 50 mg/kg/day exhibited inhibitory effects on tumor growth in a breast cancer model. In this specific instance, piperine was delivered at a concentration that exceeded the intratumoral injection by a factor of 10. The distinctive pharmacokinetic pathway that piperine takes after consumption is responsible for this phenomenon.

In this work, the researchers employed the 4T1 cell line, which is recognized for its tendency to migrate to many organs, including the lung. The primary objective was to investigate the inhibitory effects of piperine on metastasis in an in vivo experimental model. The treatment of mice with piperine at the highest dosage investigated (5 mg/kg) led to a notable decrease in the generation of lung metastases, hence suggesting the potential of piperine as an antimetastatic drug based on in vitro investigations. There exists compelling evidence that the anticancer qualities of piperine are substantiated by in vitro data, which demonstrate its possession of antioxidant and proapoptotic capabilities, as well as antimetastatic properties.

## Conclusions

A wide range of alkaloids have exhibited effectiveness in suppressing the proliferation of cancer cells. Approval has been obtained by the FDA for the utilization of a wide array of naturally occurring alkaloid-based pharmaceuticals with anticancer properties. Chemoprevention, a crucial approach in cancer prevention, involves the utilization of phytochemicals derived from fruits, vegetables, and spices. Piperine has received much interest in contemporary times due to its chemopreventive qualities. Piperine exhibits a range of potential chemopreventive properties, such as the ability to inhibit angiogenesis, decrease self-renewal of CSCs, alter ER stress and autophagy, and impact the Wnt/-catenin and PI-3K/Akt signaling pathways. Nevertheless, the therapeutic efficacy of piperine is constrained by its hydrophobic properties and inadequate solubility in aquatic environments. The findings obtained from this study demonstrate that piperine possesses the ability to selectively target diverse cancer cell types and manifest discrete mechanisms of action contingent upon the specific cancer type. The mechanism by which piperine functions as a chemopreventive agent in inhibiting cancer formation remains a subject of ongoing discussion and analysis. To advocate for the use of piperine as a therapeutic intervention, it is crucial for researchers to get a full understanding of its precise mechanism of action. There is a potential and encouraging possibility that piperine, a chemical considered to be safe for human use, could exert a significant chemopreventive effect.
